# Adapt or avoid

**DOI:** 10.7554/eLife.14345

**Published:** 2016-02-23

**Authors:** Ganesh K Kumar

**Affiliations:** Department of Medicine, University of Chicago, Chicago, United Statesgkumar@medicine.bsd.uchicago.edu

**Keywords:** hypoxia, glutamate, synapse, signal transduction, membrane transport, <i>C. elegans</i>

## Abstract

An enzyme called p38 MAP kinase helps nematodes to adapt to low-oxygen environments, and also to escape from them.

**Related research article** Park EC, Rongo C. 2016. The p38 MAP kinase pathway modulates the hypoxia response and glutamate receptor trafficking in aging neurons. *eLife* **5**:e12010. doi: 10.7554/eLife.12010

**Image** The nematode *C. elegans* provides a relatively simple system for studying how hypoxia affects neurons

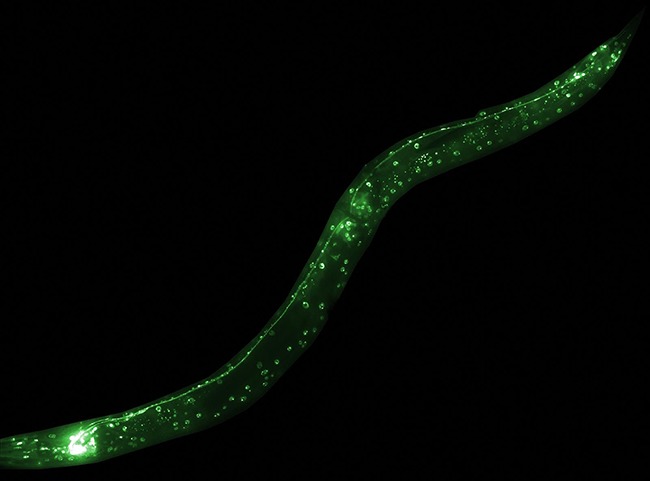

All aerobic organisms need oxygen to survive, so they need to be capable of sensing when the amount of oxygen reaching their cells and tissues becomes dangerously low. To cope with hypoxia, organisms activate various protective mechanisms that depend on the duration and/or severity of the oxygen shortage. Understanding how these mechanisms work to detect and combat environments that could deprive an organism of oxygen is a major challenge for researchers.

A large family of enzymes collectively known as protein kinases adds phosphoryl groups to proteins to regulate a wide variety of physiological processes including gene expression, protein activation and protein trafficking. Now, in eLife, Eun Chan Park and Christopher Rongo of Rutgers The State University of New Jersey report that p38 mitogen-activated protein (MAP) kinase is a key component of hypoxic response pathways in neurons of the soil-living nematode, *Caenorhabditis elegans* (Park and Rongo, 2016).

*C. elegans* is ideally suited to unraveling the secrets of how neurons protect themselves against hypoxia because its nervous system is rather small, consisting of a total of 302 neurons (White et al., 1986). Additionally, the organization of the nervous system is well-established, as are the molecular pathways that regulate neural circuitry (Macosko et al., 2009). Several toolkits are also available that allow the effects of genetic changes to *C. elegans* to be easily investigated.

*C. elegans* responds robustly to changes in oxygen levels (Carrillo and Hallem, 2015). Like in other multi-cellular organisms, the hypoxic response in *C. elegans* begins with the inhibition of the oxygen sensor EGL-9. This sensor adds hydroxyl groups to proteins that contain the amino acid proline, so when it is inhibited, a transcription factor called HIF-α (hypoxia-inducible factor α) is less likely to be hydroxylated. This, in turn, increases the expression of the genes necessary for adapting to a low-oxygen environment.

Uniquely, hypoxia also induces a behavioral response in *C. elegans.* In an environment that contains ambient oxygen levels, the nematodes display a random walk pattern of movement, with frequent reversals in direction during long runs of forward motion. However, when they encounter a persistent low-oxygen environment, the frequency with which the nematodes reverse direction is reduced. As a result, the random walk is replaced with a roaming form of motion that allows the nematodes to escape from the low-oxygen environment. This response requires a variant of the EGL-9 oxygen sensor, called EGL-9E, but it does not require HIF-α. The neurons that control locomotion in *C. elegans* contain a receptor protein called GLR-1 at their synapses. To sustain the random walk behavior, GLR-1 recycling and trafficking to the synaptic region must be maintained.

Studying both normal worms and several “loss-of-function” mutants, Park and Rongo demonstrate that p38 MAP kinase signaling is an integral component of both the transcriptional responses and the behavioral changes that help the nematode to survive hypoxia (Figure 1). Hypoxia, on one hand, inhibits the phosphorylation of EGL-9 by a p38 MAP kinase, thus inhibiting the oxygen sensor and triggering the pathway by which HIF-α increases gene expression. On the other hand, inhibiting p38 MAP kinase initiates roaming-like behavior in the worms because it interferes with the trafficking and recycling of GLR-1. Park and Rongo also show that p38 MAP kinase activity declines with age even when plenty of oxygen is available, which impairs GLR-1 recycling.

**Figure 1.**
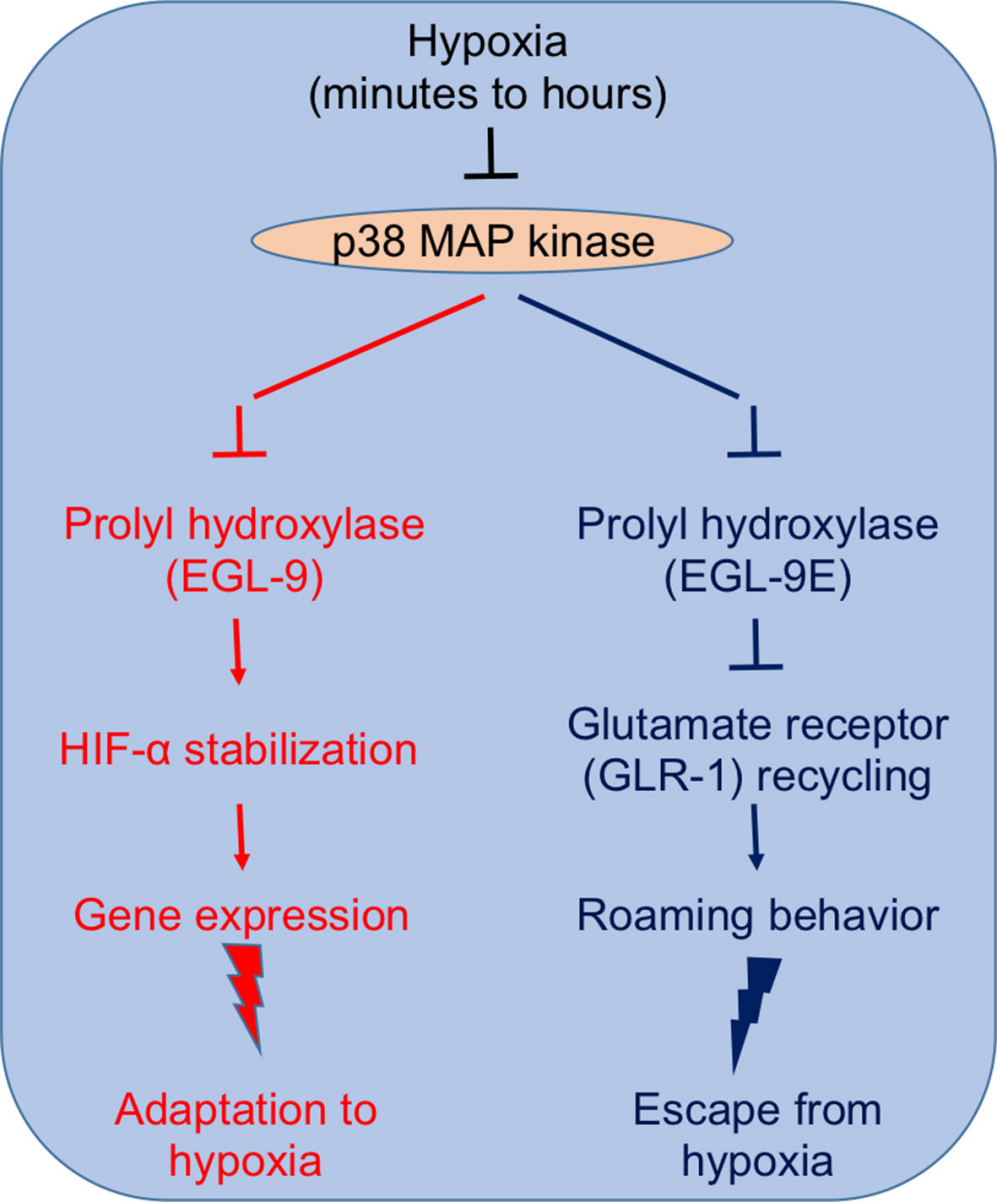
p38 MAP kinase signaling plays a central role in the responses that allow *C. elegans* to adapt to (left) and avoid (right) prolonged hypoxia (Park and Rongo, 2016). Left: Inhibiting p38 MAP kinase inhibits the activity of the oxygen sensor EGL-9. This stabilizes the transcription factor HIF-α, which promotes the expression of several genes that help the nematode to adapt to hypoxic conditions. Right: Inhibiting p38 MAP kinase also inhibits EGL-9E (which is an isoform of EGL-9). This causes EGL-9E to dephosphorylate and dissociate from a scaffold protein, which ultimately prevents the recycling of GLR-1 at the synapses of the neurons that control locomotion. The end result is that the worm starts to roam, rather than performing a random walk, which increases its chances of escaping from a hypoxic region.

Previous work investigating acute hypoxia (lasting seconds or minutes) revealed a response that relies on phosphorylation events mediated by two enzymes: soluble guanylate cyclase and protein kinase G (Gray et al., 2004; Yuan et al., 2015). The work of Park and Rongo now suggests that when hypoxia lasts for minutes or hours, varied responses are orchestrated by the EGL-9 oxygen sensor working in concert with different downstream effector molecules. Furthermore, this longer-lasting hypoxia primarily affects the phosphorylation of EGL-9E by p38 MAP kinase. However, the mechanism by which sustained hypoxia affects p38 MAP kinase activity is an open question: does sustained hypoxia directly affect protein thiol groups, which are sensitive to oxidation? Or does it have an indirect effect whereby the generation of reactive oxygen species impacts kinase activity?
